# A short, animated storytelling video about sodium intake as a major cardiovascular risk factor and recommendations for a healthy diet: an online, randomized, controlled trial

**DOI:** 10.1186/s13063-023-07418-6

**Published:** 2023-06-10

**Authors:** A Supady, K Nguyen, M Abd El Aziz, C Überreiter, T Bärnighausen, M Adam

**Affiliations:** 1 Interdisciplinary Medical Intensive Care, Medical Center - University of Freiburg, Faculty of Medicine, University of Freiburg, Freiburg, Germany; 2 Heidelberg Institute of Global Health, University of Heidelberg, Heidelberg, Germany; 3 Division of Infectious Diseases and Tropical Medicine, Heidelberg University Hospital, Heidelberg, Germany; 4 Department of Global Health and Population, Harvard T. H Chan School of Public Health, Boston, USA; 5 Africa Health Research Institute (AHRI), Somkhele, KwaZulu-Natal South Africa; 6 Department of Pediatrics, Stanford University School of Medicine, Stanford, CA USA

## Abstract

**Background:**

Increased uptake of sodium is a major cause for cardiovascular disease and mortality. Reduction of daily salt intake below a reference level of 2 g per day (the equivalent to 5 g salt/day) is known to effectively reduce cardiovascular mortality. The widespread use of social media, with a constant increase in video consumption, is opening new avenues for the dissemination of innovative and scalable approaches to health-related information and recommendations for a healthy diet, such as via video interventions with short animated stories (SAS).

**Objective:**

This study will evaluate the effect of a sodium intake-SAS video intervention on immediate and medium-term knowledge about dietary sodium. Beyond that, immediate and medium-term effects on behavioral expectation to reduce sodium intake as well as voluntary post-trial engagement with the video content will be examined.

**Methods:**

In this 4-armed, parallel, randomized controlled trial, 10,000 adult, US participants will be randomly assigned to (1) a short, animated storytelling intervention video on sodium as a cardiovascular disease risk factor followed by surveys assessing the facts on sodium and cardiovascular disease conveyed in the video (2) the surveys only, (3) an attention placebo control video followed by the before mentioned surveys, and (4) an arm that is exposed to neither the video nor the surveys. Two weeks later, participants in all four arms will complete all of the surveys.

**Results:**

Primary outcomes are the immediate and medium-term effects of the short, animated storytelling intervention video on knowledge about dietary sodium. Secondary outcomes are immediate and medium-term effects of the short, animated storytelling intervention on behavioral expectation to reduce sodium intake as well as voluntary post-trial engagement with the video content.

**Conclusion:**

This study will extend the knowledge on the effects of short, animated storytelling for the containment of the global cardiovascular disease burden. Knowledge on the groups that may be more likely to voluntarily engage with SAS video content will help to improve targeting of future interventions towards audiences at risk.

**Trial registration {2a}:**

ClinicalTrials.gov NCT05735457. Registered on February 21, 2023.

**Supplementary Information:**

The online version contains supplementary material available at 10.1186/s13063-023-07418-6.

## Synopsis/World Health Organization Trial Registration Data Set {2b}

**Table:** 

**Data category**	**Information**
Primary registry and trial identifying number {2a}	ClinicalTrials.gov (NCT05735457)
Date of registration in primary registry	February 21, 2023
Primary registry trial registration URL {2a}	https://www.clinicaltrials.gov/ct2/show/NCT05735457
Secondary identifying numbers	n/a
Source(s) of monetary or material support {4}	• The study is funded by an Alexander von Humboldt University Professor Prize awarded to Dr. Till Bärnighausen. • Dr. Alexander Supady is supported by a Berta-Ottenstein-Grant from the Faculty of Medicine, University of Freiburg.
Primary sponsor {5b}	Heidelberg University Hospital Im Neuenheimer Feld 672 69120 Heidelberg
Secondary sponsor(s)	Not applicable.
Contact for public queries	Dr. Alexander Supady, MPH University of Freiburg Medical Center Interdisciplinary Medical Intensive Care Hugstetter Str. 55 79106 Freiburg Germany Tel.: +49 761 270-73790 Fax: +49 761 270-73792 E-Mail: alexander.supady@uniklinik-freiburg.de
Contact for scientific queries	Dr. Alexander Supady, MPH University of Freiburg Medical Center Interdisciplinary Medical Intensive Care Hugstetter Str. 55 79106 Freiburg Germany Tel.: +49 761 270-73790 Fax: +49 761 270-73792 E-Mail: alexander.supady@uniklinik-freiburg.de
Public title {1}	A short, animated storytelling video about sodium intake as a major cardiovascular risk factor and recommendations for a healthy diet: an online, randomized, controlled trial
Scientific title	A short, animated storytelling video to increase knowledge about sodium intake as a major cardiovascular risk factor and to give recommendations for a low-sodium healthy diet: an online, randomized, controlled trial
Countries of recruitment {9}	United States
Health condition(s) or problem(s) studied	High-sodium diet and excess sodium intake.
Intervention(s) {11a}	Short, animated storytelling video on sodium intake
	Short, animated attention placebo control video (content unrelated to information on sodium intake)
Key inclusion and exclusion criteria {10}	Participants (all genders) aged 18-59, that have registered with the Prolific Academic platform are eligible for this trial. No further in- or exclusion criteria apply.
Study type {8}	online, randomized, controlled four parallel-arm trial
Date of first enrollment	March 1^st^, 2023 (anticipated)
Target sample size {14}	10,000
Recruitment status	Not yet recruiting.
Primary outcome(s) {12}	Immediate and medium-term effects of the short, animated storytelling intervention video on knowledge about dietary sodium.
Key secondary outcomes {12}	Immediate and medium-term effects of the short, animated storytelling intervention on behavioral expectation to reduce sodium intake as well as voluntary post-trial engagement with the video content.

## Introduction

### Background and rationale {6a}

Cardiovascular diseases are the leadingcause of death globally. According to data from the Global Burden of Disease Study, an estimated 17.8 million people died from cardiovascular disease in 2017, representing about one third of all global deaths [1]. High systolic blood pressure and an unhealthy diet are the most relevant modifiable risk factors for cardiovascular disease and are supposedly responsible for more than 10 million deaths per year [2]. Increased uptake of sodium is a major cause for elevated systolic blood pressure with a presumed linear relationship between the amount of daily sodium consumption and systolic blood pressure [3]. In a modeling study, 1.65 million deaths from cardiovascular causes, that occurred in 2010, were attributed to sodium consumption above a reference level of 2 g per day (the equivalent to 5 g salt/day) [4]. Reduction of daily salt intake has been proven to effectively reduce elevated systolic blood pressure, major cardiovascular events, and mortality [5–7]. In a recently published cluster-randomized study, conducted in China, the rates of stroke, major cardiovascular events, and death were significantly reduced in participants who lowered their sodium intake by using potassium chloride as a salt substitute, compared with the group that consumed regular salt (sodium chloride) [7]. In a large UK population-based cohort study, always adding salt to foods (as compared to never or only rarely) was linked to a 28% higher risk of premature mortality and to 1.5 and 2.28 years lower life expectancy at the age of 50 years in women and men, respectively [6]. Consequently, the World Health Organization (WHO) recommends a low-sodium diet with salt intake of less than 5 grams per day for adults. Achieving this target could help to significantly reduce premature mortality and DALYs attributed to cardiovascular disease [8].

In the typical US diet, approximately 70% of an individual’s daily sodium intake comes from eating processed food, compared with only 5.6% from home-cooked meals [9]. Therefore, in order to achieve the WHO target, key steps involve increasing knowledge in the public about which foods are high in sodium and promoting behavioral shifts that help people to reduce their dietary sodium intake.

Video-based, short, animated, storytelling (SAS) has been introduced as a novel form of entertainment-education (E-E). In recent trials, it has been proven an effective strategy for health education and the promotion of beneficial health practices [10]. E-E videos may rapidly transfer knowledge to a wide audience through popular social media channels in a cost- and time-efficient manner. For this study, we designed a short (approximately 3.5 min long), animated storytelling video to convey basic knowledge about the importance of controlling and limiting the individual sodium consumption (see multimedia Appendix). This study will systemically evaluate the effectiveness of the SAS video approach to E-E by measuring the effect of our intervention on knowledge and behavioral expectation to reduce dietary sodium intake. The result will add to the literature on SAS video health education as well as informing future E-E video approaches aimed at conveying preventive health messages. Ultimately, the results of this study may inform the future design and implementation of population-based initiatives aimed at distributing knowledge and improving behaviors in support of healthier dietary choices.

### Objectives {7}

In this study, we aim to

- Evaluate the effect of SAS videos on knowledge about dietary sodium intake in the short- and medium-term
- Evaluate the effect of SAS videos on behavioral expectation to reduce sodium consumption in the short- and medium-term
- Measure voluntary engagement with SAS video content

We hypothesize, that SAS videos help increase knowledge about dietary sodium intake in the short- and medium-term, but knowledge may decrease during the 2-week follow-up phase.

## Methods

### Trial design {8}

This study is a randomized-controlled parallel-group trial examining the effect of a short, animated storytelling video on knowledge and behavioral expectation to reduce dietary sodium intake. We will use an online academic research platform (Prolific Academic) to recruit and randomize 10,000 participants aged 18–59 years into four study groups.

In group 1 (intervention group), participants will be shown a SAS video on dietary sodium (see multimedia Appendix) and thereafter be asked to complete a questionnaire to assess knowledge on dietary sodium and behavioral expectation.

In group 2 (exposed control group), participants will be asked to complete a questionnaire to assess knowledge on dietary sodium and behavioral expectation (the same questionnaire as in group 1), however, without being exposed to the SAS video on dietary sodium before.

In group 3 (attention placebo control group), participants will be shown an attention placebo control (APC) video, unrelated to the outcomes measured in this trial, before being asked to complete the knowledge and behavioral expectation questionnaire.

Participants in group 4 (unexposed control group) will not view a video and will not be asked to complete the questionnaire.

**Fig. 1:**
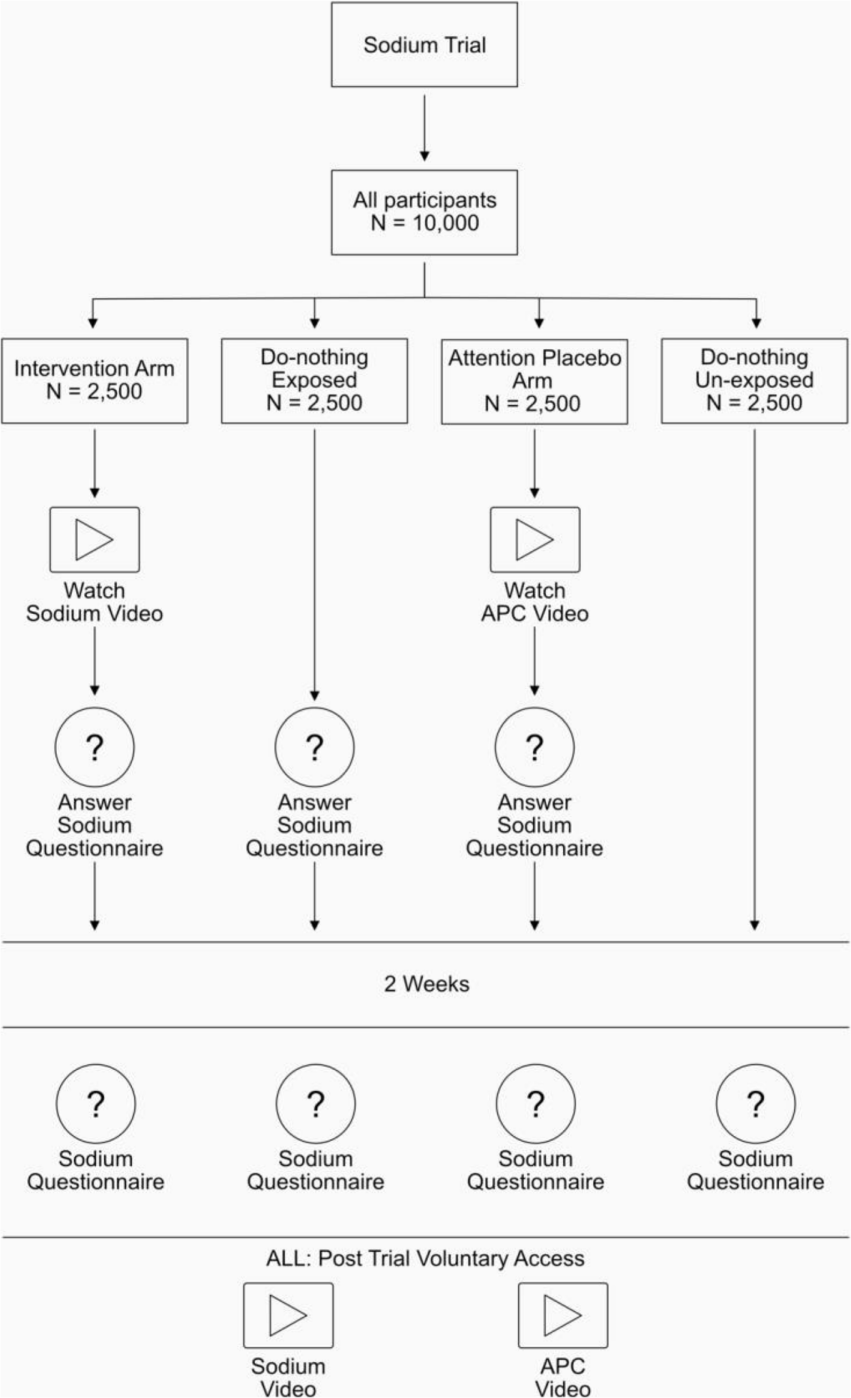
Trial design

Two weeks later, participants in all four arms will once again be asked to complete the knowledge and behavioral expectation questionnaire. Figure 1 illustrates the trial design.

### Study setting {9}

This study will be conducted purely online. Participants from the USA will be recruited and randomized through the platform Prolific Academic (ProA; https://www.prolific.co). The video intervention and questionnaires will be delivered to participants through the secure, online platform Gorilla, an experiment builder that provides users with the tools for undertaking online behavioral research (www.gorilla.sc).

### Participants and eligibility criteria {10}

ProA will recruit participants from its participant pool. Registered participants on the ProA platform must be between the ages of 18 and 59 years (all genders). For this trial, we will only recruit participants living in the USA. No further inclusion or exclusion criteria apply.

### Intervention description {11a}

The sodium intervention is a short, 2D animated storytelling video (3:09 min; https://youtu.be/QYqAbbifI9U). The video tells a humorous story about a man who dreams that his heart is threatening to quit on him. The heart character complains of being “under too much pressure” due to the amount of sodium in the man’s diet. Through this narrative, the heart character conveys foundational messages about the importance of consuming only the recommended amount of sodium, as well as how the man can change his behavior to reduce his sodium intake. The APC video is also a 2D animated storytelling video of similar length (3:29 min; https://youtu.be/LRiWQfLvQjQ) and serves as an attention placebo control. The video conveys general messages about hope and gratitude, through a fantasy story about an orange dragon who takes a group of children on a global journey. As in the intervention group, the video is shown to stimulate the subject's attention, but without conveying information related to sodium and cardiovascular risk. After watching these videos, the participants in both arms will complete a sodium questionnaire containing 30 questions and three behavioral expectation questions.

Participants can only complete the survey once.

### Explanation for the choice of comparators {6b}

At time point 1, knowledge scores and behavioral expectation will be compared between three groups that will receive the questionnaires (*Intervention Arm – Do-nothing Exposed Arm – Attention Placebo Arm*). This comparison is intended to find out the immediate effect on knowledge and behavioral expectation about sodium intake by watching the intervention video. The primary comparison will be between the *Intervention Arm* and the *Do-nothing Exposed Arm.* By comparing these groups with the *Attention Placebo Arm* we will also be able to eliminate a potential generalized attention-activation effect from watching an animated short film (unrelated to sodium).

At time point 2, comparing the *Intervention Arm* with the *Do-nothing Un-exposed Arm* will allow us to detect potential medium-term effects of watching the intervention video on knowledge and behavioral expectation — participants in the *Do-nothing Un-exposed Arm* will neither have been exposed to the intervention video nor the sodium knowledge questions at time point 1. At time point 2, comparing the *Do-nothing Exposed Arm* with the *Do-nothing Un-exposed Arm* will provide information about the effect of simply asking sodium-related questions on medium-term knowledge and behavioral expectation to reduce dietary sodium.

### Outcomes {12}

As primary outcomes, we will measure the immediate and medium-term effects of the short, animated storytelling intervention video on knowledge about dietary sodium. Secondary outcomes are the immediate and medium-term effects of the short, animated storytelling intervention on the behavioral expectation to reduce sodium intake as well as voluntary post-trial engagement with the video content.

### Sample size {14}

We calculated the sample size needed for comparison of means between selected arms as detailed in the trial design. This was done independently for the primary and secondary outcomes based on the score of each questionnaire scale, upon which we selected the most conservative sample size for our study.

The sodium questionnaire consists of 8 questions, each comprising a different number of items. In total, the participants can score up to 30 points by correctly answering all of the items in the questions. As this is a new questionnaire, there is no prior information on the average score and its variability in the general population; thus, the effect size cannot be directly estimated. In addition, we assume that a consistent change in knowledge through watching an animated video could be achieved by watching the video not only once, but several times. As our study provides a one-time view of the video and measures the effect afterwards, we aim to measure a small change in the average score, presuming that these small changes could turn into larger ones by watching the video again. Thus, we power the trial to detect a small effect size for comparing the mean score between samples, i.e., *d*=0.1, smaller than the generally recommended threshold for detecting a small effect size [11]. We used the sample size formula for one-tailed comparison of the mean score with equal variance, a type I error of 0.01 to account for multiple comparisons, and a power of 80% to detect the changes in the mean score. This resulted in a sample size of 2009 individuals per arm. Accounting for a potential attrition rate of 20% (personal communication with ProA), this yields 2410 individuals per arm. For this study, we will recruit 10,000 participants (2500 per arm), which allows us to detect the potential changes in the intervention with a power higher than 80%.

### Recruitment and strategies to improve adherence to interventions, promote participant retention, and complete follow-up {15, 11c, 18b}

Adequate participant enrolment to reach the target sample size will be guaranteed by Prolific Acedemic (ProA). ProA has a sufficiently large database of registered prospective trial participants.

The major strategy to improve adherence to the interventions in this trial is to offer payment to all participants. The payments will be given only after completing the whole survey (i.e., once the medium-term follow-up questionnaire is finished). If participants spend more than 45 min on any phase of the trial, they will be timed out of the experiment. This prevents participants from burdening the system with partially completed questionnaires. Throughout the study, investigators will have no access to the identity of the participants, so follow-up with individual participants is not possible. All incomplete datasets from participants who discontinue or deviate from intervention protocols will be descriptively reported.

### Sequence generation, allocation concealment, and implementation: {16a, 16b, 16c}

This 4-arm randomized controlled online study will be hosted and implemented by the *Gorilla experiment-builder platform*. The platform will use a computer-generated allocation sequence to randomly allocate participants 1:1:1:1 to the four trial arms. There is no human involvement, and the process is fully concealed from both study investigators and prospective participants until the study arm is assigned. The investigators will remain unaware of individual trial arm allocations.

### Blinding {17a and 17b}

All study investigators and researchers involved in the data analyses will remain blinded to the trial arm allocation for the entire study duration. Unblinding will not be required.

### Participant timeline: {13}

Participants will be enrolled over a 4-week period on the Prolific Academic platform. Phase 1 of the trial (video viewing and subsequent knowledge testing) will occur on a rolling basis, with Phase 2 (follow-up knowledge testing) taking place 2 weeks subsequently, across all arms. After both phases have been completed, each participant will be offered post-trial access to both videos. Their voluntary view time will be recorded as a measure of participant engagement with this health education medium (Table 1).

**Table 1:** Schedule of enrolment, interventions, and assessments

Timepoint**	**Study period**							
	**Enrolment**	**Allocation**	**Post-allocation**					**Close-out**
	***-t***_***1***_	**0**	***Intervention video (t***_***1***_***)***	***Attention placebo video (t***_***1***_***)***	***Sodium and behavioral expectation questionnaire (t***_***1***_***)***	***2-week interval***	***Sodium and behavioral expectation questionnaire (t***_***2***_***)***	***Post-trial video access (all videos)***
**Enrolment:**								
**Eligibility screen**	X							
**Informed consent**	X							
**Allocation**		X						
**Interventions and assessments:**								
***Intervention Arm***			X		X	X	X	X
***Do-nothing Exposed Arm***					X	X	X	X
***Attention Placebo Arm***				X	X	X	X	X
***Do-nothing Un-exposed Arm***						X	X	X

### Relevant concomitant care permitted or prohibited during the trial {11d}

There are no restrictions regarding concomitant care during the trial.

### Criteria for discontinuing or modifying allocated interventions {11b}

Participants may withdraw their consent to participate in the study at any time before or during the study. Data from incomplete surveys will not be used in the primary analysis, except at a meta-level to report the aggregate number of incomplete surveys.

### Provisions for post-trial care {30}

During this trial short, animated storytelling videos will be displayed and questions related to the health effects of sodium intake and behavioral expectations regarding sodium consumption will be asked. The trial will be conducted purely online.

There are no foreseeable risks associated with participating in this study. Participants volunteer and consent to participate in the study and can withdraw at any time. The authors of the study can be contacted at any time after the study, should the participants wish to follow up.

### Plans for assessment and collection of outcomes: {18a}

The Gorilla platform will collect data submitted by individual participants as they select their responses to the knowledge and behavioral expectations questions. We anticipate completing all data collection within a 4-week window.

### Data management {19}

Gorilla will store all data on their secure cloud platform, hosted on Microsoft Azure in the Republic of Ireland. Encryption of the Gorilla database is ensured by industry-standard cryptography, however, the research team controls and maintains ownership of all data. The research team can access only anonymized data sets and, for statistical analysis purposes, we will download and safely store this data on secure computers maintained by our co-investigators at Heidelberg University, Germany.

### Statistical methods for analyzing primary and secondary outcomes {20a}

#### Descriptive statistics

Descriptive statistics will be used to summarize the distribution of the measured scores by demographic factors, including factors such as gender, age, region, political affiliation, socio-economic status, ethnic, and minority, since these factors could affect the scoring.

#### Primary outcomes

For each participant, the overall score achieved in the sodium questionnaire will be calculated individually. The mean score for each study arm at time point 1 and time point 2 (i.e., re-assessment 14 days after entering the trial) will be calculated and compared between the *Intervention Arm*, the *Attention Placebo Arm*, and the *Do-nothing Exposed Arm* at time point 1 and between all four study groups at time point 2, using the *t*-test for two independent groups. We will also use multilevel regression analyses to control for demographic factors and other potential variables as needed. The analyses will be conducted using the R statistical software package (R 4.2.2).

#### Secondary outcomes

For evaluation of secondary outcomes, we will use the statistical methods described above to evaluate both the immediate and medium-term effects of the SAS video intervention on behavioral expectations to reduce sodium intake. The methods include, but are not limited to, *t*-test for independent groups, matched t-test for dependent groups, and multilevel regression model. For that, we will analyze the mean scores of the Sodium questionnaire at time points 0 and after 14 days, with a focus on the impact of the SAS video intervention (“Intervention Arm”) on the score outcomes. We will use the program R 4.2.2 for the statistical analyses.

### Methods for additional analyses (e.g., subgroup analyses): {20b}

To quantify the participants’ engagement, we will use the graphical experiment builder in Gorilla to give participants an option to rewatch the video before ending the study. We will record a timestamp when the participant reaches the end of the second time point, the moment when they play the videos, and the moment when they end the study. Participants who skip the video or watch it for less than 3 seconds are considered as not engaging. For the participants who watch the video, we will record the length of time spent watching the videos. We will use regression models to describe the effects of sociodemographic factors and the Sodium questionnaire score with engagement time.

### Methods in analysis to handle protocol non-adherence and any statistical methods to handle missing data {20c}

Participants in the study will have a time limit of 45 min to watch the short, animated storytelling videos and answer the questionnaires. All items of each questionnaire are designed such that all questions have to be answered before the questionnaire can be submitted. This will guarantee that there will no missing values in the datasets of participants included in the analyses. All participants who have participated and completed the first round of the study (time point 1) will be included in the study of medium-term effect (time point 2, 14 days later). If a participant, who submitted a completed survey at time point 1, fails to complete a survey at time point 2, their data will only be included in the immediate effect analysis. Participants who do not watch the videos or answer the questionnaire items at either time point will be excluded from the analysis.

## Ethics, oversight, monitoring, and dissemination

### Ethical approval and protocol amendments {24, 25}

Ethical clearance for the conduction of this study was obtained from the Stanford University Institutional Review Board (IRB) on June 7th, 2022 (protocol #65160) and a modification to this protocol was approved on Feb. 6th, 2023. Any further modifications to the study protocol will be communicated to the Stanford IRB office by the study team in a timely manner. Changes to the study protocol will not take effect before approval by the Stanford IRB.

### Informed consent {26a, 26b}

The process of obtaining informed consent will be conducted by the ProA platform. Prior to their enrolment into the study, each participant must give their informed consent, in which they will be thoroughly informed about the purpose, potential benefits, and potential risks of participating in the study. Participants will also receive contact information for both the principal investigator and the Stanford Ethics Review Board, should they have any concerns. Individuals who register on ProA are required to agree to the terms and conditions for collection and use of participant data, as well as the privacy policy of ProA.

### Confidentiality {27}

All participants will remain anonymous to the investigators for the entire duration of the trial. At no point in time, will the investigators have access to identifying information of the participants. All participants will be assigned unique ID numbers, no conclusions can be drawn to individual participants. If participants choose to contact the study team, they will be informed that their names may become visible to the study team. The study team commits to keeping all correspondence confidential.

### Access to data {29}

All investigators listed above will have access to the full and complete data set. There are no contractual agreements with any other party with respect to collection, evaluation or use of the data within this trial.

### Data monitoring and trial steering committees {21a, 21b}

This study will be overseen by a trial steering committee (TSC) made up of an independent chairperson, further members, and the study collaborators. At least two TSC meetings will be scheduled during the planning and data collection periods.

Due to the minimal risks related to this study and the very short study period, no additional data monitoring committee will be required. For the same reason, no interim analyses are planned and no specific stopping rules will be put in place.

### Adverse event reporting and harms {22, 23}

This is a minimal-risk trial. As such, we do not anticipate any adverse events. The TSC will monitor trial conduct during data collection.

### Dissemination plans {31a, 31b}

We anticipate the data collection to be completed by the end of May 2023. We seek to publish the trial findings in peer-reviewed journals and aim for presentations at scientific conferences. Authorship will be considered according to ICMJE criteria.

### Plans to give access to the full protocol, participant-level data and statistical code {31c}

This document is the full protocol. Anyone interested in other data or documentation should contact the corresponding author. The R code for the analyses will be published together with the planned publications on Github.com, as appropriate.

## Discussion

As a fully online, randomized controlled trial, this protocol describes an innovative approach to public health education in general and, more specifically, to the prevention of cardiovascular disease. Sustainable dietary sodium reduction hinges on knowledge about which foods contain high levels of sodium and how to reduce dietary sodium through behavioral shifts. As such, interventions that improve knowledge and catalyze behavioral shifts in the public offer a compelling additional tool for reducing the burden of cardiovascular disease.

While designing this trial, we considered the fact that our participant population may not be representative of the general US population. Prior research has found that users registered on ProA tend to be slightly younger and slightly more educated than US averages [12]. However, the benefits of using ProA include access to a large, geographically dispersed, adult US test population, with minimal loss to follow-up. We conclude that, since the intervention described in this protocol, if effective, would eventually be delivered via online platforms, testing it with an online community is reasonable and perhaps even advantageous.

## Trial status

Protocol version number and date: Version 1.0, 28.02.2023

Recruitment status: Not yet recruiting

Estimated study start (recruitment) date: March 1, 2023

Approximate study completion (end of recruitment): May 31, 2023

### Supplementary Information

**Additional file 1.** Sodium Knowledge Questionnaire.

## Data Availability

Anyone interested in other data or documentation should contact the corresponding author. The R code for the analyses will be published together with the planned publications on Github.com, as appropriate.

## Appendix

Intervention video: https://youtu.be/QYqAbbifI9U

APC: https://youtu.be/LRiWQfLvQjQ

## Abbreviations

SAS: Short, animated storytelling
E-E: Entertainment-Education
ProA: Prolific Academic
APC: Attention Placebo Control
TSC: Trial Steering Committee
IRB: Institutional Review Board
