# Supplementary material for: A short, animated storytelling video about sodium intake as a major cardiovascular risk factor and recommendations for a healthy diet: an online, randomized, controlled trial
Source: Trials. 2023 Jun 10;24:390. doi: 10.1186/s13063-023-07418-6 (PMC10257297; doi:10.1186/s13063-023-07418-6)
Supplement: Supplementary file 1 — Additional file 1. Sodium Knowledge Questionnaire. [file 13063_2023_7418_MOESM1_ESM.docx]

Sodium Knowledge Questionnaire

HIGH-Freiburg-Stanford

1. Which of the following is **the best** way to reduce the sodium in a typical US diet?

a) reduce the amount of processed food eaten at each meal

b) limit the use of the salt shaker by removing it from the table at each meal

c) eat more breads and cereals at mealtimes to “crowd out” salty foods

d) substitute red meats (like steak) with fish and seafood (like shrimp) at mealtimes

e) all of the above are effective ways of reducing sodium in the typical US diet

2. Which of the following foods are *significant contributors of sodium to a typical diet* in the US?

**A. salty snacks** **(like chips and pretzels)**

(choose “yes” if you think this food is a significant contributor and “no” if not)

a) yes

b) no

**B. shrimp (frozen, non-breaded)**

(choose “yes” if you think this food is a significant contributor and “no” if not)

a) yes

b) no

**C. chicken drumsticks (raw, fresh)**

(choose “yes” if you think this food is a significant contributor and “no” if not)

a) yes

b) no

**D. deli-style turkey slices**(choose “yes” if you think this food is a significant contributor and “no” if not)

a) yes

b) no

**E. canned soup**(choose “yes” if you think this food is a significant contributor and “no” if not)

a) yes

b) no

**F. breakfast cereals**(choose “yes” if you think this food is a significant contributor and “no” if not)

a) yes

b) no

**G. canned vegetables**(choose “yes” if you think this food is a significant contributor and “no” if not)

a) yes

b) no

**H. fried chicken**(choose “yes” if you think this food is a significant contributor and “no” if not)

a) yes

b) no

**I. fresh vegetables**(choose “yes” if you think this food is a significant contributor and “no” if not)

a) yes

b) no

**J. packaged bread**(choose “yes” if you think this food is a significant contributor and “no” if not)

a) yes

b) no

**J. packaged bagels**(choose “yes” if you think this food is a significant contributor and “no” if not)

a) yes

b) no

**K. fresh fruit**(choose “yes” if you think this food is a significant contributor and “no” if not)

a) yes

b) no

**L. cheese**(choose “yes” if you think this food is a significant contributor and “no” if not)

a) yes

b) no

**M. pizza**(choose “yes” if you think this food is a significant contributor and “no” if not)

a) yes

b) no

**N. instant ramen noodles**(choose “yes” if you think this food is a significant contributor and “no” if not)

a) yes

b) no

**O. plain pasta (no sauce)**(choose “yes” if you think this food is a significant contributor and “no” if not)

a) yes

b) no

**P. restaurant hamburgers**(choose “yes” if you think this food is a significant contributor and “no” if not)

a) yes

b) no

**Q. frozen vegetables**(choose “yes” if you think this food is a significant contributor and “no” if not)

a) yes

b) no

**R. sauces and condiments**(choose “yes” if you think this food is a significant contributor and “no” if not)

a) yes

b) no

3. True or False? Sodium is the same thing as salt in the diet.

a) true

b) false

4. What percentage of table salt is made up of sodium?

a) 2%

b) 10%

c) 25%

d) 30%

e) 40%

f) 50%

g) 70%

h) 100%

5. High sodium consumption contributes to which of the following health problems in the US?

**A. high blood pressure**
(choose “yes” if you think high sodium contributes to this problem and “no” if not)
a) yes

b) no

**B. high blood sugar**
(choose “yes” if you think high sodium contributes to this problem and “no” if not)

a) yes

b) no

**C. high blood cholesterol**

(choose “yes” if you think high sodium contributes to this problem and “no” if not)
a) yes

b) no

**D. cardiovascular disease**
(choose “yes” if you think high sodium contributes to this problem and “no” if not)
a) yes

b) no

**E. strokes**

(choose “yes” if you think high sodium contributes to this problem and “no” if not)
a) yes

b) no

**F. arthritis**

(choose “yes” if you think high sodium contributes to this problem and “no” if not)
a) yes

b) no

6. What is the maximum recommended daily intake of sodium?

a) 5,000mg

b) 2,300mg

c) 3,400mg

d) 1,800mg

e) 4,500mg

7. What is the maximum recommended daily intake of salt?

a) ¼ tsp

b) 1.5 tsp

c) ½ tsp

d) 1 tsp

e) 2 tsp

8. What percentage of daily sodium intake comes from processed foods in a typical US diet?

a) 10%

b) 25%

c) 30%

d) 50%

e) 70%

f) 90%
